# Cognitive Mapping Without Vision: Comparing Wayfinding Performance After Learning From Digital Touchscreen-Based Multimodal Maps vs. Embossed Tactile Overlays

**DOI:** 10.3389/fnhum.2020.00087

**Published:** 2020-03-17

**Authors:** Nicholas A. Giudice, Benjamin A. Guenther, Nicholas A. Jensen, Kaitlyn N. Haase

**Affiliations:** ^1^Spatial Informatics Program: School of Computing and Information Science, The University of Maine, Orono, ME, United States; ^2^Virtual Environments and Multimodal Interaction (VEMI) Laboratory, The University of Maine, Orono, ME, United States; ^3^Department of Psychology, The University of Maine, Orono, ME, United States

**Keywords:** wayfinding without vision, cognitive mapping, haptic displays, accessible digital maps, blind navigation

## Abstract

This article starts by discussing the state of the art in accessible interactive maps for use by blind and visually impaired (BVI) people. It then describes a behavioral experiment investigating the efficacy of a new type of low-cost, touchscreen-based multimodal interface, called a vibro-audio map (VAM), for supporting environmental learning, cognitive map development, and wayfinding behavior on the basis of nonvisual sensing. In the study, eight BVI participants learned two floor-maps of university buildings, one using the VAM and the other using an analogous hardcopy tactile map (HTM) overlaid on the touchscreen. They were asked to freely explore each map, with the task of learning the entire layout and finding three hidden target locations. After meeting a learning criterion, participants performed an environmental transfer test, where they were brought to the corresponding physical layout and were asked to plan/navigate routes between learned target locations from memory, i.e., without access to the map used at learning. The results using Bayesian analyses aimed at assessing equivalence showed highly similar target localization accuracy and route efficiency performance between conditions, suggesting that the VAM supports the same level of environmental learning, cognitive map development, and wayfinding performance as is possible from interactive displays using traditional tactile map overlays. These results demonstrate the efficacy of the VAM for supporting complex spatial tasks without vision using a commercially available, low-cost interface and open the door to a new era of mobile interactive maps for spatial learning and wayfinding by BVI navigators.

## Introduction

Throughout the years, there have been many studies suggesting that people who are blind or visually impaired (BVI), particularly those with early-onset and total blindness, exhibit a range of spatial deficits on spatial learning and wayfinding behaviors as compared to their sighted peers (for reviews, see Golledge, 1993; Millar, 1994; Thinus-Blanc and Gaunet, 1997; Ungar, 2000; Long and Giudice, 2010; Schinazi et al., 2016). Although there is some debate, what can be summarized from this body of literature suggests that BVI individuals tend to perform well on egocentric spatial tasks in small-scale “local” environments, i.e., pointing from their position to another location in a room, or on learning and navigating known routes. However, under-developed spatial skills and performance errors are often cited in the literature with this demographic when learning and navigating larger, unfamiliar environments (e.g., buildings, campuses, neighborhoods, cities, et cetera). The skills needed for successful environmental learning and wayfinding of these large-scale spaces go beyond the perception of one’s immediate environment and the following of known routes. Accurate performance requires complex sensorimotor couplings and spatio-cognitive processes that employ allocentric spatial knowledge (understanding spatial relations of the environment independent of one’s current position and heading in the space), spatial inference (such as determining shortcuts or detours from known routes), spatial updating (understanding how self-to-landmark and landmark-to-landmark relations change as a function of movement), and developing accurate survey (map-like) knowledge of global environmental relations (Golledge, 1999; Montello, 2005).

Maps represent an excellent tool for supporting many of these critical wayfinding behaviors in large-scale environments that cannot be directly perceived, such as aiding in determining ones current location or planning routes during pre-journey or *in situ* navigation (Montello et al., 2004), and for developing allocentric mental representations of global spatial structure, called cognitive maps (O’Keefe and Nadel, 1978). Although most maps are visual in nature, there is a long history of tactile map use by BVI individuals (Perkins, 2002). Indeed, tactile maps represent an excellent solution for the spatial challenges often ascribed to BVI people as they provide a means of conveying access to off-route landmarks and global spatial structure that is simply not possible to apprehend from nonvisual environmental sensing. We hypothesize that increased availability of tactile maps, accompanied with better formal instruction on how they should be read and used, would greatly improve environmental awareness, spatial inference, and cognitive map development for BVI wayfinders, thereby mitigating many of the spatial deficits that have been ascribed to this demographic in the literature. The two incarnations of accessible maps studied here (a hybrid interactive map and a haptic touchscreen-based digital interactive map) are aimed at meeting this need, each with different pros and cons, as described below.

## Background

### Traditional Tactile Maps

The importance of tactile maps on BVI spatial learning and cognitive map development has strong empirical support in the literature. When comparing tactile map learning and direct experience with blind adults, Espinosa et al. (1998) found that over multiple trials and with delays between training and test, tactile map learners were much better on measures of both route and survey knowledge than participants who learned only from direct environmental experience, showing that access to tactile maps led to improved cognitive map development in terms of accuracy and flexibility. Similar benefits on cognitive map development have been shown with BVI people using tactile maps to learn novel spaces (Golledge, 1991; Ungar et al., 1997a) and for BVI travelers using tactile maps while actively navigating the environment (Blades et al., 1999). Access to maps is especially important for BVI children, as in addition to the benefits found with adults, their use promotes the development of spatial thinking and spatial problem solving (Ungar et al., 1995, 1997b).

Despite the many benefits of tactile maps on spatial learning and navigational performance, they also have several shortcomings that have greatly limited their availability and usage by both BVI children and adults. Most of these issues relate to the map authoring and production process or to limitations of the tangible output. Traditional tactile maps consist of raised elements conveying spatial properties (points, lines, and regions), surface attributes conveying symbolic properties and feature characteristics (dots/dashes, texture variation, and line-height/thickness), and braille labels to convey feature names or semantic information (for reviews, see Edman, 1992; Rowell and Ungar, 2003b). The authoring process for effectively converting visual maps into tactile analogs or developing these materials from scratch involves specialized human expertise, which is expensive in terms of both time and labor costs. Once authored, tactile maps (and other graphical content) are traditionally rendered using specialized, purpose-built, and expensive equipment, such as tactile embossers that produce dots (much like Braille) on hardcopy paper media, raised output produced on thermoform plastic sheets, or tactile output produced on heat-sensitive microcapsule swell paper (Rowell and Ungar, 2003a). Beyond these authoring and production costs, tangible maps/models are limited in that the accessible information provided is static (i.e., presented from a fixed perspective which does not change in register with the observer’s movement), cannot be updated if the underlying information changes without re-authoring/production of the map, and the output (often containing many pages of large hardcopy maps) can be cumbersome to carry/use during *in situ* navigation.

### Interactive Maps

An alternative to static tactile maps are solutions based on digital displays that convey interactive map information. These displays generally provide context-sensitive information about the navigator’s position and orientation on the map through a combination of tactile and auditory feedback. Interactive Digital maps have many benefits and address many of the inherent shortcomings of traditional static tactile maps and models. Some advantages include: (1) they are dynamic vs. static; (2) they can be multimodal vs. just tactile; (3) they can be implemented on portable platforms vs. requiring large-format sheets or map booklets; (4) they are able to be produced on commercial hardware [e.g., the Vibro-audio maps (VAM) rendered using smart tablets studied in this article] vs. expensive, purpose-built hardware; and (5) they support spatial and querying operations that are simply not possible with physical maps (e.g., map panning, zooming, and scrolling operations, where am I queries, and search functionality). Projects developing and evaluating accessible digital maps vary widely in the technology employed and in what nonvisual information is used. A good categorization of this technology, given by Ducasse et al. (2018), distinguishes between “Hybrid Interactive Maps (HIMs)” based on a physical tactile map overlaid on a digital map *via* a touch-sensitive surface and “Digital Interactive Maps (DIMs)” based solely on a digital map.

### Hybrid Interactive Maps (HIMs)

One of the earliest incarnations of an accessible digital map was Nomad, a HIM system that incorporated a traditional tactile map overlaid on a digital tablet that provided auditory information about routes and landmarks (Parkes, 1988, 1994). As with most HIM approaches, Nomad worked by registering every x-y location of the tactile map with the corresponding position on the underlying digital map. This allowed for anywhere that the user touched on the tactile map to be augmented with a speech-based label/description (or any auditory information) to be triggered at that location or region. Throughout the years, several other systems have adopted this multimodal audio-tactile HIM approach, with results showing clear benefits for supporting pre-journey route learning, spatial knowledge acquisition, and global environmental understanding by BVI people (Holmes et al., 1996; Jacobson, 1998; Landau and Wells, 2003; Kane et al., 2013). The clear advantage of HIMs is that they afford access to a traditional tactile map but with all the benefits of an underlying digital map. On the one hand, this approach would seem to represent the best of both worlds of accessible map production. On the other hand, the tactile overlays require careful authoring and specialized hardware to produce, the registration process of the overlay to the underlying digital map can be slow and cumbersome, and if the overlay is moved or shifted during use, the correspondence between the physical and digital map is lost. In addition, the use of large touch-sensitive surfaces limits portability and mobile usage. If accessible maps are to be useful tools for BVI navigators *in situ*, as is the case for their sighted peers, then these factors cannot be ignored.

### Digital Interactive Maps (DIMs)

An alternative to HIMs are haptic DIMs, representing accessible digital maps with no physical map overlay. These systems generally include some form of haptic information coupled with auditory cues as the user interface (UI) and an underlying digital map. The obvious advantage of the DIM approach is that they are self-contained, with no need for external tactile maps or physical media to be registered with the digital map. Multiple haptic technologies have been used in these interactive maps, including force-feedback devices, refreshable pin-arrays, and vibrotactile displays (see O’Modhrain et al., 2015 for a thorough review). Recent DIMs, as are studied in this article, are even able to be rendered on commercial, mobile hardware, meaning they have significantly lower cost and greater portability than is possible from other interactive mapping solutions.

#### Force Feedback DIMs

These devices work by providing differential positive/negative forces to the hand or arm to indicate points, edges, and regions of a digital 2D or 3D map/model or by employing differential friction and elasticity information to simulate a material texture or compliance (O’Modhrain et al., 2015). Research with these systems addressing accessible interactive maps has employed either commercial force-feedback devices, such as a haptic mouse or joystick, or a robot arm or manipulandum, such as the PHANTOM, which tends to provide a larger active workspace, more force, and access to 3D renderings but at the cost of greater expense and less portability. Crossan and Brewster demonstrated that the combination of force feedback from a PHANTOM Omni device, with sound feedback and a tactile pin array for specifying the direction, was effective in promoting the learning and navigation of virtual mazes by 10 BVI users (Crossan and Brewster, 2006). In a study comparing learning of maritime environments between traditional tactile maps vs. virtual maps combining auditory sounds/labels and force-feedback using a Phantom device, six totally blind participants showed equivalent accuracy in triangulation of landmark configuration after exposure to both types of maps (Simonnet et al., 2011). Highly similar performance for describing topological relations by five BVI people was also demonstrated after learning an indoor multi-room environment with a traditional tactile map vs. a virtual simulation explored using a commercial Logitech force feedback joystick or mouse, with the most analogous results found with the haptic mouse (Nemec et al., 2004). These studies provide important evidence for the efficacy of multimodal, interactive maps as performance is directly compared with traditional hardcopy tactile maps (HTM), with favorable (i.e., highly similar) results observed after learning from haptic DIMs. These results are in agreement with several other projects utilizing force feedback devices to study haptic learning of maps and virtual environments by BVI users, with results generally being positive in terms of cognitive map accuracy and user preference, e.g., The BATS project (Parente and Bishop, 2003), Open Touch/Sound Maps (Kaklanis et al., 2013), and the Haptic Soundscape project (Jacobson, 2004; Golledge et al., 2005; Rice et al., 2005). Despite these demonstrations, DIMs relying on force-feedback devices are not portable and more importantly, are limited in the information they are able to convey. For instance, they generally rely on a single point of contact (e.g., the Phantom) and while these devices are excellent for conveying surface information (e.g., texture, compliance), force-based interfaces are far less amenable to supporting accurate line tracing and contour following as other DIMs (O’Modhrain et al., 2015), which is problematic as these are critical exploratory procedures for haptic map exploration.

#### Dynamic Pin Array DIMs

This technology works by individually actuating a matrix of small pins, similar to braille dots, that can be made to move up and down to create dynamic tactile points, lines, regions, and other spatial elements needed for haptic perception of tangible maps (or any visual graphic more generally). Several projects have demonstrated the efficacy of this technology, usually combined with multimodal (audio) cues. Zeng and Weber (2010) showed that audio and haptic information delivered *via* a large, page-sized pin-matrix array (BrailleDis 9000), coupled with an OpenStreetMap (OSM) GIS database, promoted apprehension and pre-journey learning of a university campus by four blind users. The same authors also showed that access to portable audio and tactile-pin matrix system supported accurate understanding of you-are-here symbols during real-time navigation of a north-up OSM street layout by eight BVI participants (Zeng and Weber, 2016). Use of an 8-pin, mouse-like display was also shown to be effective for learning of digital Maps and diagrams in a study with 17 BVI users, especially when combined with an intelligent zooming technique employing intuitive transitions between functional levels of information presentation, rather than traditional stepwise zooming (Rastogi et al., 2013). While dynamically actuated pins have the advantage of providing excellent cutaneous feedback—a hallmark of traditional tactile maps (something that is missing with force-feedback devices and touchscreen-based displays), they involve many moving parts and are expensive to produce and maintain, which has limited their broad deployment. In addition, while there are many types of actuators used in such displays, most are not commercially available and those that are, e.g., the HyperBraille, can cost up to $50,000 for a large pin-matrix display suitable for rendering maps and large graphical content (Russomanno et al., 2015).

#### Touchscreen-Based DIMs

Touchscreen-based smart devices represent the most recent and fastest growing technology for authoring/rendering digital maps. There are many benefits of these devices for use as an accessible mapping solution. For instance, contrasting with any other technology supporting interactive maps, phones/tablets are built around a portable form factor and computational core that is inherently multi-use, multi-sensory, and incorporates many out-of-the-box universal/inclusive design features in the native interface that benefit BVI users (e.g., screen reader, magnification, and gesture interactions). Perhaps most important, these devices are based on commercial hardware that is inexpensive compared to specialized solutions and is estimated to already be in the hands of 70–80% of BVI cell phone users (WebAIM, 2015; Palani et al., 2016).

With respect to accessible map using, Timbremap was one of the first projects to show that users could learn an indoor layout by exploring a phone’s touchscreen with their hand while receiving combinations of speech messages and auditory sound cues to specify the map information, as assessed by the accuracy of subsequent verbal route descriptions (Su et al., 2010). Another system, called Tikisi For Maps, showed that 12 BVI teens could effectively learn and navigate layered map information and perform complex map scaling operations based on audio and speech cues given during exploration of a tablet’s touchscreen (Bahram, 2013). Although search performance was varied, Kane et al. (2011) showed that 14 BVI participants could learn the spatial relations of auditory targets *via* bimanual exploration of a large interactive table-top touchscreen.

### Touchscreens and Haptic Interactions

A growing number of studies have gone beyond only using auditory/speech information on the phone/tablet by also leveraging the device’s built-in vibration motor to provide haptic (vibrotactile) output. While information access is still performed by the user moving their finger around the device’s touchscreen, haptic effects are triggered by vibrating the device whenever their finger contacts an onscreen visual element. These vibrations provide robust focal stimulation to the finger, leading to the perception of feeling tactile points, lines, and regions on the touchscreen (Giudice et al., 2012). An obvious shortcoming of this approach is that unlike the traditional reading of tactile maps, there is usually only one point of contact (generally the dominant index finger) when exploring the touchscreen and there is no direct cutaneous stimulation on the finger—the user is just touching a flat glass surface. This means that many of the explicit tactile cues used with traditional embossed tactile maps (or dynamic pin-array systems) are not directly specified using vibrotactile displays, i.e., immediately perceiving a line’s orientation, thickness, and elevation (Klatzky et al., 2014). These attributes can be specified *via* vibrotactile cuing, but doing so requires active hand movement and a slower extraction process (Giudice et al., 2012).

We posit that these limitations are more than offset by the positive attributes afforded by the use of vibrotactile information. For instance, tactile information does not mask other potentially useful (or dangerous) environmental cues during real-time navigation, as is often the case when using audio/speech only displays. In addition, in contrast to the other haptic DIM approaches discussed here, the creation of touchscreen-based DIMs allows for the combination of vibrotactile and audio information using portable, inexpensive commodity hardware and does not require the time and effort to produce and register a tactile overlay with the underlying digital map (e.g., HIMs). Finally, the combination of vibrotactile cues, auditory information, and kinesthetic feedback from hand movement on the touchscreen provides more useful information about a map than is possible from touchscreen-based audio-only maps. Indeed, as haptic information (including vibrotactile cuing) is most similar to visual sensing for encoding and perceiving spatial information (as is critical to map using), touch has been argued as the preferred nonvisual analog for conveying spatial data (Giudice, 2018). Support for this claim comes from both behavioral studies and neuroimaging research showing the similarity of spatial representations built up after learning from vision and touch. For instance, functionally equivalent performance on spatial updating tasks has been found after learning haptically or visually encoded route maps (Giudice et al., 2011) and an fMRI study demonstrated that the same brain region, called the Parahippocampal Place Area (PPA), was similarly innervated by spatial computation of scenes apprehended through haptic and visual perception (Wolbers et al., 2011). Explanations for these findings of common performance between modalities, coupled with the same neural basis of action, have been explained by models from several theorists. At their core, all of these models argue that the information learned from separate inputs is stored in unified “amodal” spatial representations in the brain that can be accessed and acted upon in a functionally equivalent manner when supporting subsequent spatial behaviors, e.g., the Spatial Image (Loomis et al., 2013), the metamodal brain (Pascual-Leone and Hamilton, 2001), or the spatial representation system (Bryant, 1997).

#### Haptic Touchscreen-Based DIMs

Given these clear advantages, there are a growing number of studies utilizing touchscreen-based smart devices for providing BVI users with audio-tactile access to many types of graphical content (for general reviews, see Grussenmeyer and Folmer, 2017; Gorlewicz et al., 2019). With respect to interactive multimodal maps, TouchOver map was an early project showing that eight blindfolded-sighted users could accurately reproduce an OSM-based road network learned from a touchscreen-based map rendered with vibrational cues indicating roads and auditory labels providing street names (Poppinga et al., 2011). A study using purely vibrotactile cues also showed accurate learning of simple street maps with six BVI users as explored from both a phone and a watch touchscreen interface (Grussenmeyer et al., 2016). In a study using an early version of the VAM touchscreen-based DIM interface evaluated here, nine blindfolded-sighted participants were found to be as accurate in learning an indoor hallway layout with the VAM as with a traditional tactile map, as assessed by both pointing and map reproduction tasks (Raja, 2011). Research investigating rendering of large format maps that extend beyond a single tablet display has also demonstrated that VAM-based DIM interfaces are effective for supporting both nonvisual panning and zooming operations. For instance, accurate learning of simulated indoor maps requiring nonvisual panning techniques to learn the entire spatial extent was shown by 6 BVI users on egocentric pointing and map recreation tasks (Palani and Giudice, 2017), and by performance on similar tasks by 12 blindfolded-sighted participants found after learning VAMs requiring nonvisual map zooming (Palani et al., 2016). Adopting a slightly different approach, the GraVVITAS project demonstrated that floor-plan maps could be accurately understood by six BVI users who explored a touch tablet with multiple vibration motors attached to their fingers (Goncu and Marriott, 2011) and the SpaceSense project showed that 12 BVI users could accurately learn spatial relations of a street network using a 3 × 3 grid of external vibration motors mounted in a case on the back of an iPhone (Yatani et al., 2012). The advantage of these external vibration systems is that haptic cuing could be given to multiple digits and triggered at different regions of the screen when touched, rather than relying on only one finger and a single vibration motor (i.e., the limitation of available commercial hardware). The downside of using external vibration motors is that it requires the purchase of additional hardware and software coordination with that hardware, thereby increasing system cost and design complexity, which will inevitably reduce adoption by BVI end-users compared to systems based on unmodified commercial hardware. There is obviously a trade-off of many factors when designing new technologies but given the specific challenges of the prohibitive cost, limited usability, and low adoption for many assistive technology projects, we argue that it is most important to develop solutions with the highest probability of actually reaching the target end-user for whom it will most benefit. As there is already significant penetration of smart devices by BVI users, we believe it is most fruitful to develop solutions that can leverage all the existing advantages of this technology without requiring any additional hardware. We also feel strongly in the principle of utilizing as much multimodal information as possible from auditory, haptic, and kinesthetic channels, even if redundantly specified, as both empirical and user preference results from multiple touchscreen-based mapping studies support the benefit of multimodal vs. unimodal interfaces. For instance, the development of cognitive maps was more accurate (and less effortful) when the digital maps were learned by 12 BVI users with a combination of vibration and audio feedback vs. only audio information (Yatani et al., 2012). Similar empirical/preference benefits for combined haptic and audio multimodal touchscreen interfaces vs. their unimodal analogs have been shown for map learning with 14 BVI participants on comprehension of indoor layouts (Adams et al., 2015), with 6 BVI and six blindfolded-sighted user’s on map recreation tasks after learning the relation between three landmarks on a tablet-based digital map (Simonnet et al., 2019) and with map learning by 12 BVI users *via* a small touchscreen-based watch interface (Bardot et al., 2016). In aggregate, these studies demonstrate the value of using multimodal information for learning maps *via* the touchscreen, and germane to the current study, show that the use of vibrotactile information is particularly important for supporting cognitive map development and is most preferred as a mapping interface by users.

## Experiment and Methods

The current study addresses environmental learning, cognitive mapping, and wayfinding performance by blind and visually impaired (BVI) participants in unfamiliar indoor layouts (floors of university buildings). The study was designed to directly address two key gaps in the extant literature on accessible digital maps.

(1) Comparison between DIMs, vs. hybrid interactive maps (HIMs): most research with interactive tactile maps has used one or the other of these techniques but has not directly compared them using a within-subjects design and testing procedure that explicitly probes cognitive map development and wayfinding performance. This study evaluates learning with a VAM, which is a DIM that is rendered using vibrotactile and auditory information and is explored on the touchscreen of a commercial tablet vs. learning by exploring a HIM comprised of a traditional HTM overlaid on the same touchscreen and augmented with the same auditory cues. This comparison is important as the VAM-based DIM is implemented on commercial touchscreen-based smart devices and does not require the cost/effort associated with production and registration of additional tactile maps, i.e., the HIMs approach. Results showing that learning from the VAM supports similar performance as is found with the HTM-based HIM would open the door for a new class of DIMs that can be easily rendered and broadly deployed on what has become the fastest-growing computational platform used by BVI individuals.
(2) Perceptual vs. cognitive focus: Most of the research discussed above, irrespective of the technology used to render the digital maps, has focused on perceptual factors relating to map reading (e.g., information extraction/encoding). When cognitive factors were addressed, they related to how well the map information was learned and represented in memory. Common performance metrics included map reconstruction, distance and direction estimates, route following, and answering spatial questions. Most studies assessed map learning through non-ambulatory spatial tasks (but see Zeng and Weber, 2016) and did not probe the relation of how map-learning impacted cognitive map development. While cognitive maps were assessed through map reproduction tasks, this approach does not speak to how well those cognitive maps can be subsequently accessed and used during *in situ* navigation, i.e., the gold standard metric for determining efficacy for actual usage. Here, we adopted an environmental transfer technique that allows us to directly evaluate cognitive map accuracy by comparing wayfinding performance after learning maps rendered using a DIM vs. a HIM on a common testing protocol done from memory (i.e., without access to the map). With this approach, virtual representations of the two physical environments are first learned through free exploration using the two accessible mapping conditions. After the learning phase, participants are brought to the corresponding physical environment and asked to perform wayfinding tasks, such as target localization, route finding, or spatial inference. Since no explicit routes were specified during map exploration, and the map used during learning is not present at the test, successful *in situ* wayfinding behavior requires accessing an accurate cognitive map built up during the learning process. Similar transfer paradigms have proven effective for supporting accurate navigation in large physical indoor layouts after learning in the corresponding virtual space using verbal and auditory interfaces (Giudice and Tietz, 2008; Giudice et al., 2010; Connors et al., 2014). To our knowledge, the transfer paradigm has never been used to study cognitive mapping and wayfinding behavior after learning large-scale environments using touchscreen-based DIMs. However, a study by Brayda et al. (2018) has investigated small-scale environmental transfer using a refreshable pin-based dynamic tactile display. In their study, 10 BVI participants learned a map conveying a scaled representation of a physical 6 × 4.5 m room and a virtual target either *via* a static tactile map or from a dynamic 12 × 16 pin array display. After haptic learning, participants first recreated the map and then were brought to the physical room and asked to walk to the target location. Results showed that access to the dynamic map at learning led to lower errors on map recreation and faster, more accurate, and greater confidence during subsequent physical room navigation. While this study did not compare a haptic DIM with a HIM, i.e., the tactile map control was static, and the environment was much smaller than is studied here, we believe that its findings, in conjunction with results from the transfer studies in large-scale environments with auditory displays, suggest that learning from our interactive map displays will lead to accurate wayfinding performance at test. Further, building on the success of previous studies evaluating touchscreen-based haptic DIMs for map learning, discussed above, we predict that the use of the VAM-based DIM at learning will be as effective as learning from the tactile map overlay (HIM) in cognitive map development and subsequent wayfinding behavior.

### Participants

Eight blind participants, four females and four males (ages 18–55, *SD* = 13.9), were recruited for the study (see Table 1 for participant demographics). All participants were daily iPhone users and had received at least 10 h of formal orientation and mobility training. The study was approved by the University of Maine’s IRB and all participants were given informed consent and were paid for their time.

**Table 1:** Descriptive information for blind participants is provided for the following factors: etiology of blindness, age of blindness onset, residual vision (if any), level of education (high school = HS, undergraduate degree = UG, and graduate degree = G), primary mobility aid, and days per week of independent navigation.

Etiology of blindness	Onset age	Residual vision	Education	Mobility aid	Ind. Nav. Days
Diabetic retinopathy	29	N	UG	Cane	2
Microphthalmia	Birth	N	UG	Dog	7
Retinitis pigmentosa and glaucoma	Birth	Avoid obstacles	UG	Cane	7
Posterior polymorphs dystrophy, cataracts and glaucoma	Birth	N	UG	Dog	2
Lebers congenital amaurosis	Birth	Light/dark, blurry objects, avoid obstacles	UG	Dog	7
Genetic neurofibromatosis (optic pathway tumor)	5	Blurry objects, fingers at arm’s length	HS	Cane	3
Lebers congenital amaurosis	Birth	Light/dark, blurry objects, avoid obstacles	G	Dog	7
Juvenile glaucoma	Birth	Light/dark, blurry objects, avoid obstacles, fingers at arm’s length	UG	Cane	7

### Environments

**Figure 1:**
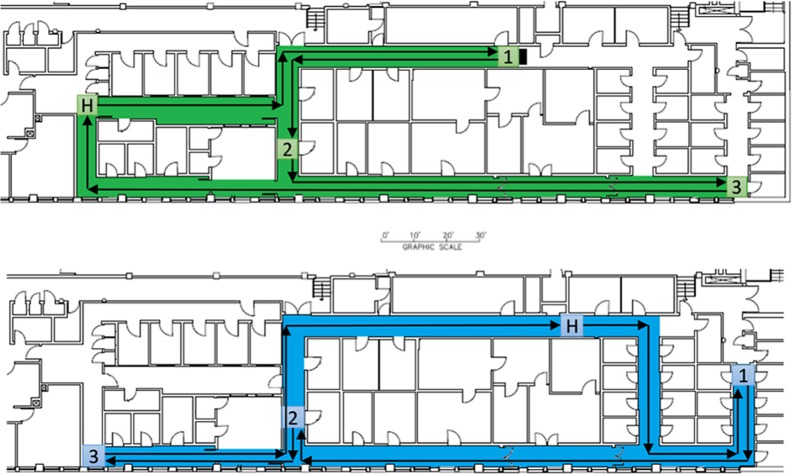
Building floorplan and maps for the two experimental conditions. The highlighted region of the floorplan was selected for the map. The four, square regions in each map represent the three objects and home locations of the maps. The arrows illustrate an example of optimal route efficiency for successful wayfinding (start and end at H).

Three virtual indoor map layouts were created based on partial floor plans within two buildings at the University of Maine. The practice map included a section of the first floor of Boardman Hall and the two experimental maps included sections of the third floor of Little Hall. The experimental maps/building layouts varied in overall topology but were matched in terms of their complexity. The experimental maps (and associated physical layouts) were similar in size, e.g., the overall corridor length of the navigable space was 398 and 411 ft (121.3 and 125.3 m) and both layouts consisted of 3 two-way intersections, 2 three-way intersections, two dead ends, and a loop (see Figure 1). All junctions were 90° and all participants were unfamiliar with the testing environments prior to the experiment. Each map contained a home location and three unique targets (map 1: doll, cat, knife; map 2: duck, carrot, shoe). Target names were selected from an index of highly visualizable and readily memorable words (Snodgrass and Yuditsky, 1996). The target name was spoken *via* synthesized speech triggered whenever its x-y location on the map was touched during the learning period. The target and home locations were selected to ensure they were spread evenly throughout each layout as well as to provide multiple walking paths between each object, thus allowing us to assess optimal and sub-optimal route-finding performance at test.

### Apparatus

**Figure 2:**
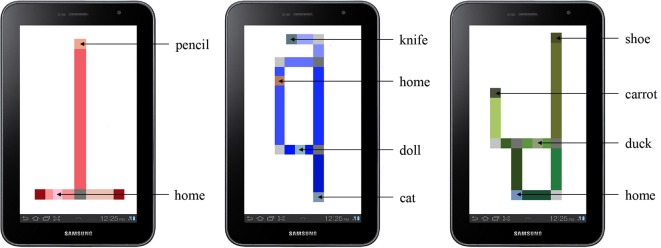
The practice map (left) and experimental maps (center and right) as rendered on the tablet. The home and target object locations are labeled.

**Figure 3:**
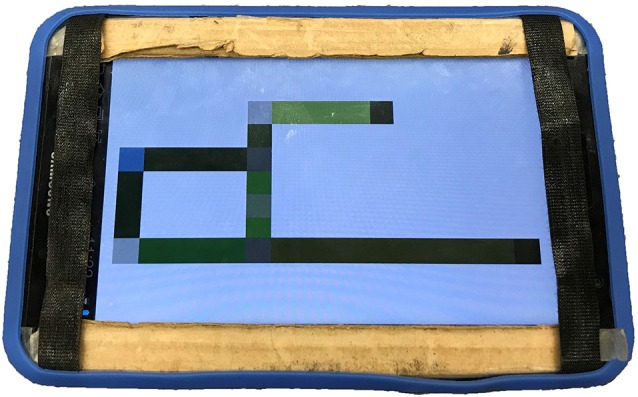
Photograph of the vibro-audio map (VAM)-based Digital Interactive Map (DIM) as used by participants.

Maps were presented *via* a Samsung Galaxy Tab (GT-P2610) with a 7.6” × 4.8” (19.3 × 12.2 cm) screen running Android 3.2 Honeycomb. Following earlier work in our lab, each map consisted of lines [0.35 in (0.9 cm)] on the tablet which represented walkable hallways (see Figure 2) and squares [0.35” × 0.35” (0.9 × 0.9 cm)] which represented objects and hallway junctions (Giudice et al., 2012). When a user’s finger touched a map element, the interface provided information *via* text-to-speech audio labels. Spoken elements included key features of the map such as hallway junctions: “corner,” “three-way,” or “dead-end,” or target objects: “cat.” Users could tap on any part of a hallway to hear the total length of the corridor between junctions (in feet). The spoken target labels and home location could also be repeated by tapping on its x-y position on the map. Volume was user selected, and the speech played at a rate of approximately 150 words per minute. The edges of the screen were framed using cardboard (see Figure 3) to provide a haptic border and to eliminate accidental contact with any “soft” buttons of the device that could interfere with the map presentation software. Maps were presented *via* either the VAM interface (DIM) or a HTM that was mounted on the same tablet (HIM), allowing for identical auditory cues between conditions and equivalent logging of finger movement behavior.

The VAM interface, representing a haptic DIM (see Figure 3) provided user feedback in the form of a continuous vibration when the user’s finger touched a walkable hallway and a pulsed vibration when the user’s finger touched an object or the home location. Contact with a walkable hallway produced a steady, 250 Hz vibration. Contact with objects and hallway junctions produced a pulsed vibration consisting of a 100 ms pulse (50% duty cycle). Use of pulsing to differentiate map/graphical elements and to draw the user’s attention to that location has been found to be important for information extraction and interpretation using vibrotactile stimuli in other studies using similar non-visual touchscreen interfaces (Giudice et al., 2012; Klatzky et al., 2014).

**Figure 4:**
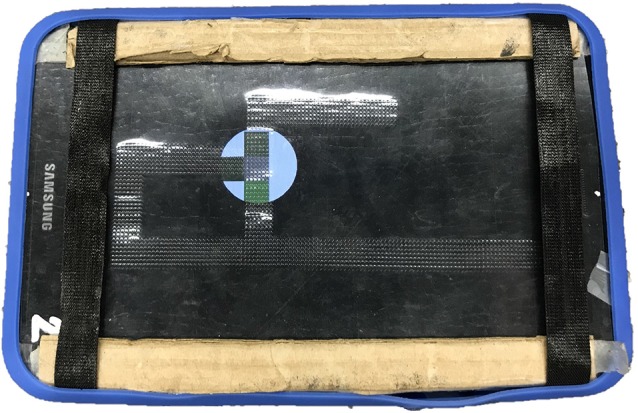
Photograph of the Hybrid Interactive Map (HIM) tactile overlay as used by participants.

The HTM overlay, representing a haptic HIM (see Figure 4), provided embossed tactile lines of the corridors instead of vibrotactile lines. These lines were produced using a View Plus Tiger Emspot embosser with 20 DPI resolution. The audio cues and functions (e.g., tap for hallway length and repeating of audio labels) with the HIM were identical to those in the VAM condition. As traditional tactile maps do not generally use (or need) additional cues to indicate vertices or intersections, as has been found for VAMs (Giudice et al., 2012), they were not included on the HTM-based HIM. However, to provide a redundant cue for targets, both map modes employed an alert tone that was triggered at that location prior to the speech message. As such, although the maps were not strictly identical, they were functionally matched in terms of all relevant information based on the rendering modality. Participants were blindfolded during map learning in both conditions to eliminate the chance of unintended effects of vision for people with any residual sight.

### Design and Procedure

A 2 (VAM-based DIM vs. HTM-based HIM) × 2 (two experimental map layouts) mixed factorial design was employed. Each participant ran in two conditions, including learning both layouts, using both map interfaces (one for each layout), and performing wayfinding tests in both physical environments. We intended for interface and map layout to be counterbalanced but due to a balancing error, five participants started with the DIM and three started with the HIM (thus the design was not fully counterbalanced). The experiment took between 75 and 120 min for each participant.

#### Practice Phase

Each participant began with a practice phase which started with an explanation of each map interface. They were first given examples of the stimuli to familiarize themselves with how each would feel. The sample HTM (which would be familiar to most BVI people) was given and then a sample practice map was provided using the DIM. They were also encouraged to practice haptic scanning strategies using the DIM, with several exploratory procedures demonstrated based on evidence of effective strategies found in earlier studies from our lab (Giudice et al., 2012; Palani and Giudice, 2017). One exploratory procedure involves back and forth sweeping of the finger across the vibrating line. Another strategy was to trace a circle around an encountered junction to determine how many legs (i.e., hallways) were intersecting at that decision point.

During the formal practice session, participants were given up to 5 min to freely explore the practice map, which consisted of a single object and two hallways (see Figure 2). Participants were instructed to inform the experimenter when they felt confident in their understanding of the spatial layout of the map as well as the location of the object within the environment. After initial exploration, the practice map was removed and a spatial memory task was administered as a learning criterion test. For this test, they were instructed to indicate the target location using a blank map. To perform the task, participants were provided a sheet of cardstock on which the borders of the tablet screen were represented by an embossed line. An embossed square [0.35” × 0.35” (0.9 × 0.9 cm)] was provided that corresponds to the home position on the map. They were then instructed to indicate the approximate location of the target object within the map by placing a finger at that location (which was then marked on the paper by an experimenter). Participants were considered to have passed the criterion test if they were able to (1) correctly identify the target object (or multiple targets for the experimental conditions) by name; and (2) place the named target(s) on the paper such that the relative positions between each target and the start location maintained the correct global spatial relation (topology) of the original map. If participants did not pass this spatial memory criterion test, they were to be allotted an additional 5 min of learning time before reattempting the test (no participants required more than one learning period to pass).

Participants were then led (blindfolded) to the real-world location they had studied using the practice map and were positioned at the location corresponding to the “home” location from the map. The blindfold was removed, and they were then instructed to walk to the location where they believed the target object was located (the object was not physically present). The trial concluded when the participant informed the experimenter that they were standing at the physical location where they believed the target object had been located on the map.

Note that the practice phase was designed to clarify the experimental procedure, and to ensure any procedural based issues were not present in the experimental trials. Due to time constraints, the full practice session only occurred using the DIM, which was a completely novel interface as compared to traditional tactile maps. If problems were to arise with the procedure, they were more likely to manifest with the unfamiliar VAM-based DIM and we wanted to make sure that such issues were fully resolved before the experimental trials. As such, the practice was done with the DIM to ensure participants understood the task and could effectively use (and were comfortable with) the novel interface. It is possible this may have introduced a slight advantage for the DIM compared to the HIM. However, considering that our overall goal was to evaluate the effectiveness of the DIM to support wayfinding behavior, this possibility was deemed acceptable for the present research. Upon accurate completion of the practice phase, participants were guided (blindfolded) to the third floor of Little Hall to begin the two experimental conditions.

#### Experimental Phase

##### Learning Task

Participants completed the map learning task while seated, with the tablet placed in front of them. They were allowed to adjust the position of the tablets based on personal preference. Prior to the learning tasks, all participants were blindfolded. The learning phase began immediately after the experimenter placed the participant’s dominant index finger on the “home” position on the map.

Learning was self-paced, with participants given up to 10 min to freely explore the experimental maps. This time limit was based on the average exploration time established during pilot testing. They were instructed to find the three objects (in addition to the home location), learn the global spatial layout of the map, and to remember where the three target objects were located within the map. Although they knew that they would be required to find routes between targets at test, explicit route information was not given during the learning phase. The learning period ended either when participants informed the experimenter they felt confident in their learning, or when 10 min had elapsed.

The learning criterion test was then administered requiring correct placement of the three targets (as described in the “Practice Phase” section). All of the participants met the criterion after the first learning period.

#### Environmental Transfer Phase

After meeting criterion, participants were led (blindfolded) to the corresponding real-world floor layout, and positioned at the same “home” location, in the same orientation, as was used during map learning. They were then asked to lift their blindfold and were given a target name to find in the environment. The targets were not physically present in the environment. During this wayfinding task, they were instructed to walk at their normal speed to the given object location using the most efficient route possible and to stop and verbally name the target once they believed they had reached its location (e.g., “I am now at the carrot”). Access to the digital map or any indication of the targets in the environment was not provided during testing, meaning that correct wayfinding performance required accessing an accurate cognitive map in memory built up from previous map learning. Testing occurred without blindfold as we were interested in the ability to access the learned cognitive map to perform wayfinding tasks, not in participant’s mobility skills for using their cane or dog guide to detect environmental features (which are very different skills). As such, to ensure that all BVI wayfinders had access to the same layout information, and to avoid any potentially confounding biases from individual differences in mobility performance, the experimenter verbalized basic corridor intersection information when encountered during the route-finding task. Thus, whenever the participant entered an intersection, they were provided a consistent verbal prompt structured to match the information provided during learning (i.e., “three-way,” “corner,” or “dead-end”). This information was provided so participants could focus on the navigation task of interest, rather than mobility challenges of identifying the intersection geometry. The experimenter did not give any information about where to go at the intersection or other cues about the current location or target position, meaning that users had to independently plan/execute their walking trajectory. After a successful navigation attempt, participants were then asked to navigate to the next object. In this manner, the participant navigated to the first object, a second object, third object, and then a return to the home location. If the participant incorrectly localized the target, they were informed they made an error and were walked (blindfolded) to the correct target location, where they once again lifted the blindfold and proceeded with the next navigation trial. This corrective procedure ensured that errors did not accumulate between route trials. Two experimenters accompanied the participant through this *in situ* navigation phase. One supervised the participant, prepared the route by opening doors and blocking unused halls, and recorded navigation time *via* a stopwatch. The other experimenter recorded the participants’ route and their response for each target location on a printed floorplan.

### Statistical Analysis

Our goal in this research was to evaluate the use of a new commercial DIM, called a VAM as a robust alternative to traditional HTM overlays on digital maps (e.g., HIMs). Based on the efficacy of previous research with similar Vibro-audio interfaces, our hypothesis was that learning with the VAM-based DIM used here would demonstrate functionally equivalent wayfinding performance as was observed after learning from the information-matched HIMs. When the *a priori* goal of hypothesis testing is to not find an effect between conditions, i.e., not reject the null hypothesis, the use of traditional frequentist based null hypothesis significance testing (NHST) is less meaningful, as these procedures allow for the determination that data are unlikely given that the null hypothesis is true (Raftery, 1995), but they do not provide evidence in support of the null hypothesis (which is the goal of the present research). An advantage of the Bayesian approach is that it provides an opportunity to analytically determine whether the null hypothesis is more likely than the alternative(s) given the observed data (e.g., Raftery, 1995; Gallistel, 2009). As such, the current data was analyzed using Bayesian methods. Although these procedures require more effort, we believe they are better suited for our purposes and that this is the first use of this rigorous (equivalence) analysis with wayfinding data in the BVI literature.

## Results

The effect of the user interface [i.e., learning a building layout *via* a DIM (touchscreen-based VAM) vs. a HIM (touchscreen-based HTM)] was evaluated using three dependent variables (DVs): (1) wayfinding accuracy; (2) route efficiency; and (3) learning time. Wayfinding accuracy was recorded as a binary variable (0 or 1) indicating failure/success for each target localization trial during the wayfinding task. Each route navigation trial was recorded as correct if the participant stopped within a 12 feet (3.7 m) radius of the target location. This accuracy threshold was selected because it is similar to the spatial extent of a single finger width on the map. Thus, there was a natural correspondence between felt location during learning and the error allowed during navigation in the physical space when localizing the target. Route efficiency was also recorded as a binary variable indicating if, during successful wayfinding, the route navigated was either the shortest/most direct route or a longer/suboptimal route. Each of the eight navigation trials were designed so that there were two possible direct routes to the target. The most efficient route was the shortest and required the least amount of turns. The direct (but inefficient routes) included traveling a greater distance, more turns, or both. Additionally, any successful navigation trial not following any of these defined routes was scored as inefficient. This included situations in which participants changed their route mid navigation (e.g., backtracking). These types of accuracy data are often analyzed by submitting participant averages (e.g., proportion correct) to an ANOVA or *t*-test; however, accuracy data (like that in the present study) are often based on a series of binary (correct/incorrect) outcomes. As a result, there is a growing body of literature that argues it is more appropriate to use generalized linear mixed-effects probit/logit models to analyze these types of accuracy data (Dixon, 2008; Jaeger, 2008; Quené and van den Bergh, 2008; Song et al., 2017). Therefore, these data were evaluated using separate mixed-effects probit regression models to estimate the effect of the interface (DIMs vs. HIMs) on the DVs of wayfinding accuracy and route efficiency. Each model included random effects (varying intercepts) for subjects and the target during wayfinding. The effect of the interface was included as a fixed effect in each model. Initial considerations of the raw data (see Table 2), suggests that the DIM is effective as a navigation aid. These data revealed similar wayfinding accuracy using both the HIM (78%) and DIM (75%). Additionally, the route efficiency data suggest that participants were using accurate cognitive maps during wayfinding after learning with both the HIM (79%) and the DIM (67%) conditions. The primary disadvantage of the DIM over the HIM is revealed in the learning time data in which the mean learning time was 194 s for the HIM and 364 s for the DIM. This disadvantage is not surprising as the DIM has less explicit cues which may slow the learning time, albeit not the overall wayfinding performance. In the following sections, we consider the effect of interface on the DVs of wayfinding accuracy and Route Efficiency. First, we define the statistical models used in this analysis (“Bayesian Model Description for Wayfinding Accuracy and Route Efficiency” section). Then, we describe how we calculated and evaluated the posterior distribution (“MCMC Sampling” section). Next, we present our conclusions based on the posterior distribution (“Evaluation of the null hypothesis *via* HDI and ROPE” section). Finally, separate analyses for the DV of learning time are presented (“Learning time” section).

**Table 2:** Mean participant data (±1 SD) for accuracy, route efficiency, and learning time.

Interface	Accuracy (%)	Route efficiency (%)	Learning time (s)
HIM/HTM	78 (±25)	79 (±19)	194 (±111)
DIM/VAM	75 (±23)	67 (±32)	364 (±180)

*Means for accuracy and route efficiency were calculated as the mean of each participant’s average wayfinding accuracy or route efficiency*.

### Bayesian Model Description for Wayfinding Accuracy and Route Efficiency

**Figure 5:**
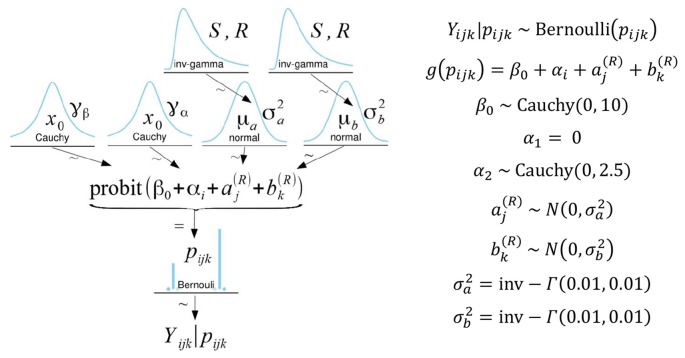
On the left is a diagram illustrating the model, priors, and hyperpriors used in the analysis. Specific model parameters are listed on the right. α_1_ is set to zero as a constraint for model identification such that α_1_ reflects the baseline condition (HIM). Therefore, α_2_represents the effect of the DIM relative to the HIM (Song et al., 2017). In the model, the subscripts i, j, and k refer to the interface, target, and participant, respectively.

For both wayfinding accuracy and route efficiency, multilevel models (see Figure 5) considered participants’ responses using a Bernoulli distribution with a probit link function. Prior distributions for the intercept and the fixed effect were assigned as Cauchy distributions using parameters recommended for weakly informative priors in logistic/probit regression (Gelman et al., 2008, 2013). Prior distributions for the random effects were assigned as normal distributions with weakly informative inverse-gamma distributions as hyper-priors for the variance parameters.

### MCMC Sampling

Bayesian analyses on the above models were conducted using the BinBayes.R function (Song et al., 2017), which uses the coda (Plummer et al., 2006) and rjags (Plummer, 2018) packages in R (R. Development Core Team, 2018) to run Markov chain Monte Carlo (MCMC) sampling *via* JAGS (Plummer, 2003) to compute the posterior distribution of the model parameters. After MCMC sampling, model convergence was first verified through visual checks of trace plots and then *via* the Gelman-Rubin convergence diagnostic (Gelman and Rubin, 1992) for each parameter using a 95% confidence interval. For all parameters, $\hat{R}$ < 1.01 indicating MCMC chain convergence. To ensure the MCMC sample was sufficiently large, the effective sample size (ESS) was calculated (Kass et al., 1998). To ensure stable estimates of the Highest Density Interval, it is recommended that MCMC sampling should be run, for parameters of interest, until the ESS for the posterior distribution exceeds 10,000 (Kruschke, 2014). The primary parameter of interest was the fixed effect of the map interface (α_2_ in the model) on both wayfinding accuracy and route efficiency. To ensure that the ESS for this parameter exceeded 10,000, the BinBayes.R function was modified in the following ways. The number of MCMC burn-in iterations was set to 5,000 and then MCMC sampling was conducted using three chains for 30,000 iterations, with a thinning interval of 5, leaving 6,000 samples per chain (total samples = 18,000). ESS exceeded the recommended value for the fixed effect both on wayfinding performance (ESS = 11,777.6) and route efficiency (ESS = 11,964.4), indicating the MCMC sample should be large enough to produce stable estimates of the HDI. The extent to which the prior informed the posterior distribution was assessed *via* the prior posterior overlap (PPO) calculated using the MCMCvis package (Youngflesh, 2018). The overlap for the fixed effect (α_2_) and intercept (β_0_) was 32.6% and 17.5% for wayfinding accuracy and 34.6% and 17.2% for route efficiency, indicating the priors did not excessively influence the posterior distribution.

### Evaluation of the Null Hypothesis *via* HDI and ROPE

Using mean values from the posterior distribution, we first calculated the marginal effect at the mean, to predict future wayfinding accuracy depending on the interface. This revealed, for an average participant, navigating to an average target, the predicted probability of successful wayfinding is nearly identical for both the HIM (82%) and the DIM (83%) conditions. This is consistent with the observed data (see Table 2) which also shows highly similar wayfinding accuracy after learning *via* the HIM (78%) and DIM (75%).

**Figure 6:**
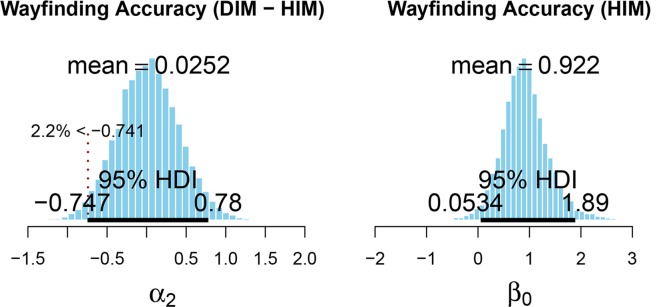
Posterior distribution for the fixed effect (left) and intercept (right) of the model for wayfinding accuracy. The lower limit of the Region of Practical Equivalence (ROPE) is indicated by the dashed vertical line and was set to correspond to −25% probability (based on the mean of the posterior for β_0_) of successful wayfinding when using the DIM compared to the HIM. This figure was created using the code provided in *DBDA2E-utilities.R* (Kruschke, 2014).

To test for equivalence, the effect of the interface (HIM vs. DIM) was assessed *via* Bayesian parameter estimation using the Highest Density Interval (HDI) plus a Region of Practical Equivalence (ROPE) decision rule (Kruschke, 2011; Kruschke and Liddell, 2018; Kruschke and Meredith, 2018). In the present research, we used a 95% HDI, which describes the range in which a measure should fall 95% of the time. The purpose of the present research is to evaluate the feasibility of the DIM-based VAM to support wayfinding ability among BVI users. Thus, we were primarily interested in testing for the noninferiority of the DIM (when compared to the HIM-based HTM). For noninferiority testing, only one side of the ROPE is emphasized (Kruschke, 2018). These accuracy data were only collected in 25% intervals (wayfinding accuracy was assessed four times for each interface); thus, the lower boundary of the ROPE was set to reflect −25% probability of accurate navigation when using the DIM compared to the HIM. As the mean predicted wayfinding accuracy for the HIM was 82%, the lower boundary of the ROPE (−0.741) corresponded to 57% accuracy. If the most credible values, from the posterior distribution of the effect of interface, predict greater than 57% accuracy (i.e., the entire HDI is greater than −0.741) when using the DIM, we could then conclude that the DIM is not inferior to the HIM (i.e., wayfinding performance is at least as good after using the DIM as after using the HIM). 97.8% of the HDI (see Figure 6) for the effect of the user interface (−0.747 to 0.78) falls above the decision criteria. These values correspond to a predicted wayfinding accuracy between 57–96% when using the DIM; however, 2.2% of the most credible values for the parameter fell below the decision criteria. Thus, we are unable to definitively confirm that the DIM is not inferior to the HIM. However, when considered alongside the HDI for the HIM (0.053–1.89), which corresponds to a predicted wayfinding accuracy between 52–97%, and given the small sample size here, these data suggest both the DIM and HIM are likely to support equivalent wayfinding performance. Another point to consider is, while the mean of posterior density for the effect of the interface (α_2_ = 0.025) is close to zero, the range of the HDI is nearly ±0.8. This is similar in magnitude to the mean of the posterior density for the intercept (β_0_ = 0.922). This point is emphasized because, although these data suggest a similarity between the DIM and HIM, there is still considerable variability in these estimates.

**Figure 7:**
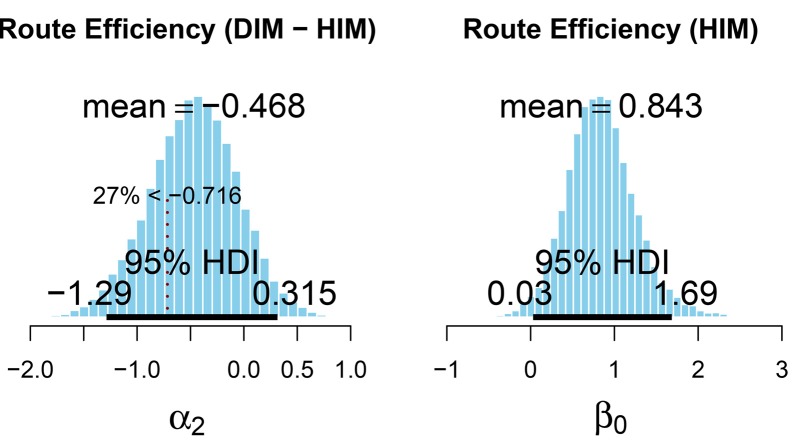
Posterior distribution for the fixed effect (left) and intercept (right) of the test model for route efficiency. The lower region of the ROPE is indicated by the dashed vertical line and corresponds to a value of −25% route efficiency (based on the mean of the posterior for β_0_) when using the DIM compared to the HIM. This figure was created using the code provided in *DBDA2E-utilities.R* (Kruschke, 2014).

The ROPE used to evaluate the effect of map interface on Route Efficiency (see Figure 7) was also set such that the lower bound (−0.716) reflected a reduced performance by 25% when using the DIM compared to the HIM. Only 73% of the HDI (−1.29 to 0.315) for the effect of interface on route efficiency was greater than the lower limit (−0.716) of the ROPE. Thus, with 27% of the HDI below the lower limit of the ROPE, these data are inconclusive regarding the effect of interface on route efficiency. Using mean values from the posterior distribution, we also calculated the marginal effect at the mean, to predict route efficiency depending on the interface. For an average participant, navigating to an average target, the predicted probability of navigating along the most efficient route is higher for the HIM (80%) than the DIM (65%). This is consistent with the observed data (see Table 2). Even though these results were inconclusive, overall route efficiency (observed data) after learning in both HIM (79%) and DIM (67%) conditions suggests participants were still using accurate cognitive maps during wayfinding. It is important to again note the variability in the parameter estimates. For route efficiency, the upper and lower values of the HDI for the effect of the interface (α_2_) deviate from the mean by approximately ±0.8. This is again similar in magnitude to the mean of the posterior density for the intercept (β_0_ = 0.843).

### Learning Time

**Figure 8:**
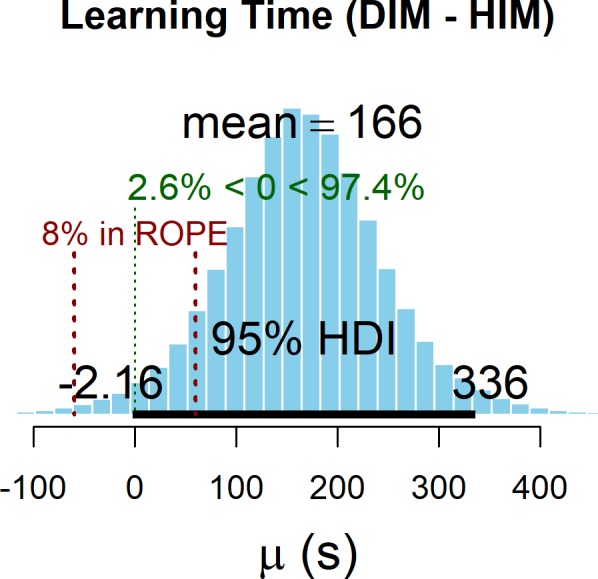
Posterior distribution for the effect of interface on learning. The ROPE is indicated in red and was set to correspond to a ±60 s of learning time. This figure was created using the BEST package (Kruschke and Meredith, 2018).

Learning time was defined as the time (in seconds) participants spent using each interface (HIM or DIM) to learn the map. The effect of interface on learning time (see Table 2) was also assessed *via* Bayesian parameter estimation using the HDI plus a ROPE decision rule. The posterior distribution was generated *via* MCMC sampling using the default options (Kruschke, 2013) in the BEST package (Kruschke and Meredith, 2018). MCMC chain convergence was indicated by an R < 1.01 for all parameters. To ensure stable estimates of the parameter of interest (mean), the ESS was confirmed to be greater than 10,000 (ESS = 45,918. Figure 8 shows the posterior distribution for the effect of interface on learning time. The positive value for the mean estimate indicates 166 s greater learning time when using the DIM compared to using the HIM. The ROPE was set such that a difference in learning time reflecting ±60 s would be considered equivalent performance. With 8% of the HDI in the ROPE, we are unable to determine whether the map interface has a reliable effect on learning time or not. However, the posterior probability that the difference in learning time is greater than zero (i.e., that it takes longer to learn using the DIM than using the HIM) was 97.4%. Thus, it is likely that the DIM requires increased learning time as compared to the HIM, an outcome that is consistent with findings comparing similar interface conditions for learning graphs and shapes (Giudice et al., 2012).

## Discussion

The goal of this experiment was to evaluate map learning and cognitive map development using a wayfinding task with BVI participants. Comparisons were made between learning with hybrid interactive maps (HIMs) consisting of traditional HTM overlays that were mounted on a touchscreen and augmented with audio information and a new class of digital-only interactive maps (DIMs) that conveyed functionally-matched information using vibratory and auditory cues *via* the same touchscreen interface. The literature has unequivocally shown that access to tactile maps greatly benefits the accuracy of environmental learning, cognitive map development, and wayfinding performance by both children and adult BVI users (Golledge, 1991; Espinosa et al., 1998; Blades et al., 1999). These advantages have also been demonstrated with accessible interactive maps, using a host of technologies (Ducasse et al., 2018). As discussed previously, there are many benefits of digital maps, e.g., they are interactive, dynamic, and multimodal as compared to traditional static, tactile-only maps but there are also challenges. For instance, most digital maps require the use of technology that is expensive, highly specialized, and non-portable. In addition, the HIMs approach still requires the use of tactile map overlays, which are costly to produce and necessitate careful registration with the underlying digital map.

The DIM we evaluated here adopted a new approach to multimodal interactive mapping, called a VAM, which is based on commercial touchscreen-based smart devices. Filling a gap in the literature, this study set forth to directly compare environmental learning with the VAM-based DIM with traditional HIMs using a within-subjects design. Rather than focusing on perceptual factors related to map reading or route following, as has been the emphasis of previous research, we adopted an experimental paradigm that evaluated map learning by measuring cognitive map development on a series of *in situ* wayfinding tasks. Our interest here was in assessing similarity between the two map learning conditions (e.g., supporting the null hypothesis), rather than the traditional hypothesis testing approach of detecting differences (e.g., rejecting the null). As such, we used Bayesian analyses aimed specifically at determining whether or not performance on a battery of wayfinding tasks between conditions was functionally equivalent. Although definitive equivalence was not observed using the strict parameters of our analyses, the consistent wayfinding data found between the two map learning conditions are indicative of a highly similar performance. This is an important outcome as it demonstrates that traditional (raised line) tactile stimuli are neither necessary for developing accurate cognitive maps nor required for supporting efficient wayfinding behaviors. While the observed data does suggest a difference in the time needed to learn the maps, with the HTM-based HIM being faster than the VAM-based DIM, these findings are not all that surprising given the nature of tactual encoding from the hardcopy stimuli, i.e., faster and more direct perception of edges and intersections compared to extraction of the same attributes using vibrotactile cues (Klatzky et al., 2014). Indeed, these results are in-line with previous literature comparing learning time between vibrotactile and traditional tactile modes across a range of spatial patterns (Giudice et al., 2012). The role of expertise is also an unknown factor that could impact learning time. While most BVI individuals have interacted with some form of traditional embossed tactile renderings during orientation and mobility training, none of our participants had previously used our VAM interface. Additional studies are needed to assess whether increased experience with the VAM leads to corresponding improvements in learning speed. The results from the navigation test are far more relevant to our interest in cognitive map development, with the current findings suggesting that once the maps are learned, the ensuing cognitive maps are similarly robust in supporting a high level of wayfinding behavior, irrespective of map learning condition.

Our study of haptic map learning between the encoding of traditional embossed tactile stimuli and vibrotactile stimuli is more than a comparison of two map presentation modes. The VAM-based DIM and HTM-based HIM also utilize different types of sensory receptors and physiological channels of haptic information processing. That is, converging results from psychophysical studies and direct physiological recordings from the glabrous skin of the human hand have identified four types of mechanoreceptors that have different spatial and temporal response properties (Bolanowski et al., 1988). While tactual perception likely involves multiple types of these mechanoreceptors and overlap of the neural substrates/channels mediating this information depending on what combinations of spatial, temporal, and thermal parameters are present, the embossed tactile maps and vibrotactile maps at the heart of our comparison utilize different fundamental receptor types. For instance, the HIM, relying on traditional mechanical stimulation from skin deformations and displacements during movement would have prioritized activation of the slowly adapting type I (SA I) and slowly adapting type II (SA II) receptors, which are most sensitive to this type of stimulation (Loomis and Lederman, 1986). By contrast, the DIM, which was based primarily on vibrotactile stimulation, would have involved the “P channel,” with Pacinian corpuscles as the primary receptor inputs. The Pacinian are sensitive to changes in thermal properties and stimulus duration but are most associated with vibration and vibrotactile stimulation between the broad range of 40 and 800 Hz (Bolanowski et al., 1988). Given that the Pacinians are most sensitive at around 250 Hz (Loomis and Lederman, 1986) and that the majority of vibration motors and actuators used in commercial smart devices operate around 200 Hz (Choi and Kuchenbecker, 2013), it is likely that our VAM-based DIM primarily activated these receptors. While more psychophysical studies are needed to formally compare the similarities and differences of haptic perception between traditional embossed tactile stimuli vs. the vibrotactile perception elicited from movement of a flat touchscreen display *via* a vibrating motor/actuator (as was the case for our VAM-based DIM), the current results provide a compelling story for how these different haptic presentation modes support real-world spatial learning. Indeed, we interpret the similarity of test performance after learning with both DIM and HIM maps observed here as supporting the notion that different encoding sources, as long as they convey functionally relevant information, can lead to a common spatial representation in the brain that functions equivalently in the service of spatial behaviors (Loomis et al., 2013). Although additional research is needed to further probe the structure of the underlying neural representation between these approaches, the current results clearly support the efficacy of the VAM as a new type of DIM interface that is comparable to traditional HIM solutions. In addition, the similarity we observed between our low-cost DIM and the traditional HIM suggests that the trade-off of additional expense and increased design complexity in producing HIMs is not justified by a corresponding offset in improved behavioral performance.

The high level of wayfinding performance observed after learning with both map conditions also provides evidence demonstrating that the use of one-finger for encoding environmental information during map exploration is sufficient for supporting accurate spatial learning and cognitive map development. These findings speak to a longstanding debate in the literature about the relevance of the use of one or more fingers in tactile perception. Clarification of this issue is particularly relevant to touchscreen-based haptic DIMs, which are generally explored by moving only one point of contact on the display, usually the dominant index finger. This exploration strategy contrasts with traditional tactile map reading, which is often done with unrestricted movement of both hands over the map. Studies using vibrotactile patterns (Craig, 1985) or embossed tactile objects (Klatzky et al., 1993) have argued for the benefit of using multiple fingers/hands. However, other research investigating the exploration of raised line drawings found no consistent benefit of using multiple fingers (Loomis et al., 1991). In two systematic studies investigating reading of simple tactile maps with multi-finger/hand use by both blindfolded-sighted and BVI participants, Morash and colleagues found that while performance tended to improve with multiple fingers, it was not a simple “more is better” scenario. For instance, while Line-tracing tasks were found to be fastest when using two hands, performance benefits were not found by using more than one finger per hand. By contrast, tasks requiring a search of both local and global structures were faster with multiple fingers, but not with both hands. Finally, BVI users tended to perform better than their sighted peers when using both hands (Morash et al., 2013, 2014). In studies employing more complex map-reading tasks, two hands were found to be beneficial but only one was employed for map exploration, while the other hand (or finger) was used as a fixed “anchor” on the map or its edge (Perkins and Gardiner, 2003). This 2-hand anchoring strategy has also been found to be useful with touchscreen-based haptic DIMs, similar to the VAM studied here, in supporting map panning operations (Palani and Giudice, 2017). Although the one vs. multiple finger issue has not been extensively studied with interactive maps, research with 14 BVI participants on learning indoor layouts *via* a touchscreen-based tablet interface using haptic cues delivered to one finger from the devices embedded vibration motor vs. stereo haptic cues delivered by vibrating rings worn on two fingers, showed that one-finger exploration was usually more accurate and actually preferred (Adams et al., 2015). The combination of these findings, in conjunction with the current results, provide compelling evidence for the efficacy of one-finger search strategies with touchscreen-based DIMS for supporting accurate information extraction, map learning, and cognitive mapping enabling efficient wayfinding behavior.

The current findings also speak to the issue of under-developed spatial abilities of BVI navigators that are often described in the literature, e.g., deficits in building up and accessing accurate cognitive maps (for review, see Thinus-Blanc and Gaunet, 1997). The consistently high wayfinding performance observed here would not have been possible without map learning leading to accurate cognitive map development. These results argue against the standard explanation of lack of vision or visual experience as being the root cause of spatial deficits by BVI people (see Schinazi et al., 2016 for discussion). We interpret our findings as supporting the information-access hypothesis of blind spatial cognition. According to this perspective, the spatial differences (if manifest) found in studies with BVI individuals compared to their sighted peers are less about the role of visual experience or a necessary outcome of vision loss but occur as a result of insufficient access to environmental information from nonvisual sensing and under-developed teaching of key spatial skills underlying complex spatial behaviors (Giudice, 2018). Maps are an excellent tool for conveying normally inaccessible environmental information; as such, they represent an important solution for leveling the “spatial” playing field for BVI wayfinders.

Finally, while visual maps are often used during *in situ* navigation, the physical limitations of traditional tactile maps (i.e., their size and cumbersome nature) and the lack of portability of most interactive mapping technologies greatly constrain analogous real-time map usage. While the small form factor of VAM-based DIM interfaces makes them particularly amenable to *in situ* use scenarios, the success of this interface in the transfer task evaluated here (i.e., map learning followed by subsequent wayfinding in the physical space) suggests that they would also be excellent pre-journey learning tools. This procedure involves exploring maps in an offline learning mode, where the map is used to learn routes, configurational information, and convey spatial relations before going to space. This strategy has been extremely effective for teaching spatial concepts to BVI navigators in a safe and low-stress scenario (Holmes et al., 1996; Zeng and Weber, 2010; Ivanchev et al., 2014). As the haptic DIMs tested here can be used for both pre-journey learning and during real-time wayfinding, they represent an important advancement in accessible mapping technology. Note that although our current findings are limited to indoor building layouts, we are confident in the VAM’s efficacy in also supporting outdoor travel, where there are far more complementary tools, technologies, and environmental cues. This prediction will be tested in a future environmental transfer study with wayfinding at test occurring in an outdoor environment.

## Data Availability Statement

The datasets generated for this study are available on request to the corresponding author.

## Ethics Statement

The studies involving human participants were reviewed and approved by University of Maine Institutional Review Board. The patients/participants provided their written informed consent to participate in this study.

## Author Contributions

NG designed the experimental tasks and contributed to the implementation of the research and to the writing of the manuscript. BG performed the data analysis and interpretation and also wrote the results sections. KH assisted with conducting the study and initial data organization and analysis. NJ assisted with piloting the research and conducting the experimental trials.

## Conflict of Interest

The authors declare that the research was conducted in the absence of any commercial or financial relationships that could be construed as a potential conflict of interest.
